# A Retrospective Study on COVID-19 Infections Caused by Omicron Variant with Clinical, Epidemiological, and Viral Load Evaluations in Breakthrough Infections

**DOI:** 10.7150/ijms.87167

**Published:** 2024-01-01

**Authors:** Jian Liu, Ru Wen, Nanzhu Wang, Guizhou Li, Peng Xu, Xiaoming Li, Xianchun Zeng, Chen Liu

**Affiliations:** 1 Department of Medical Imaging, Guizhou Provincial People Hospital, Guiyang City, Guizhou Province, 550000, China.; 2 Department of Radiology, Southwest Hospital, Army Medical University (Third Military Medical University), Chongqing, 400038, China.; 3 Medical College, Guizhou University, Guizhou,550000, China.; 4 College of Mathematics and Statistics, Chongqing University, Chongqing, 400044, China.

**Keywords:** Omicron, Viral load, Ct-value, gender, age, comorbidities, vaccination, Clinical symptoms

## Abstract

**Purpose:** To explore the clinical, epidemiological, and viral load characteristics of COVID-19 caused by the omicron variant.

**Methods:** Based on the COVID-19 epidemic caused by SARS-CoV-2 Omicron BA.2 broke out in Shanghai, China. To analyze whether there is any association between clinical symptoms and viral load of COVID-19 with age, sex, and combined disease and whether the clinical symptoms and viral load are associated with vaccine-breakthrough infections.

**Results:** The most common symptoms were cough, expectoration, and fatigue, which were more common in women than males (p < 0.001). The average viral clearance time in the > 75 years group was the longest (6.64 days). The viral load in the 60-75 years group was significantly higher than that in the other groups (p < 0.001). The 18-45 years old group had the most clinical symptoms at admission (45.39%). The days of nucleic acid-negative conversion, average viral load, highest viral load, and clinical symptoms in comorbid chronic disease patients are longer (p < 0.001). The average and highest viral loads in the unvaccinated group were longer than those in the vaccine breakthrough infection groups (p < 0.001). However, the clinical symptoms in the vaccine breakthrough infection group were significantly more severe than those in the unvaccinated group (p < 0.001).

**Conclusions:** We found that female patients, the elderly, and those with underlying comorbidities had longer clinical positive symptoms and viral loads. Although vaccination may not reduce clinical symptoms, it can shorten the viral load and the time required for virus clearance.

## Introduction

Coronavirus disease 2019 (COVID-19), caused by severe acute respiratory syndrome coronavirus 2 (SARS-CoV-2), emerged in Wuhan, China, in December 2019^1^. It is declared a global pandemic by the World Health Organization (WHO) because of its global potential and fatal outcomes. More than 600 million cases of COVID-19 and six million deaths have been reported worldwide. The Omicron variant has recently become dominant worldwide and rapidly raised serious concerns. Vaccination effectively prevents disease progression and death and is a leading strategy to change the COVID-19 pandemic worldwide 2. However, individuals of different sexes, ages, and combined diseases may produce different antibodies against the same vaccine 3. Additionally, the Omicron variant may have evolved into a significant immune escape, which shows longer transmissibility than the delta variant 4. Therefore, the SARS-CoV-2 Omicron variant may increase the risk of breakthrough vaccine infection. Despite breakthrough infections, the hospitalization rate of unvaccinated individuals with COVID-19 remains higher than that of vaccinated individuals. In addition, unvaccinated individuals have a higher risk of morbidity and mortality. Generally, COVID-19 vaccines are highly effective, especially against serious diseases 5.

Although vaccines have played an important role in reducing COVID‐19 infections, especially in reducing critically severe cases and deaths, whether vaccination can reduce the viral load and clinical symptoms has become the main topic. The type and severity of clinical symptoms of COVID-19 vary, as it can be asymptomatic, leading to respiratory failure and death due to multiple organ dysfunction. Common symptoms include fever, cough, expectoration, dyspnea, muscle pain, fatigue, loss of smell and taste, sore throat, and headache. Concomitant diseases include hypertension, diabetes, cardiovascular diseases, respiratory diseases, and malignancies. Furthermore, age and sex are important factors associated with the risk and prognosis of COVID-19^6^. However, the association of age and sex with clinical symptoms, viral load, and days to viral clearance in OmicronBA.2 has not been revealed. Especially in vaccinated people, information regarding the clinical symptoms of breakthrough infections is lacking. A thorough analysis of the infection characteristics caused by the Omicron variant is of critical importance for pandemic control, vaccine development, clinical management, public education, and international collaboration. These analyses not only contribute to a better understanding of the COVID-19 pandemic but also guide future pandemic preparedness and prevention strategies. Therefore, based on the COVID-19 epidemic caused by SARS-CoV-2, Omicron BA.2 broke out in Shanghai, China, from February to July 2022. In this study, we used 1:1 propensity score matching (PSM) to match baseline characteristics in the queue to minimize the effects of bias and confounding variables. The aim is to assess the potential associations between COVID-19 clinical symptoms, viral load, viral clearance time, and the demographic characteristics of patients, including age, sex, vaccination status, and comorbid diseases.

## Methods

### Study Design and Population

This study had a cross-sectional observational design, which included patients with mild omicron BA.2 variant infection from March 28, 2022, to June 28, 2022, in Shanghai's largest Fangcang shelter hospital. The Fangcang Shelter Hospital was designated to admit patients with mild diseases during the outbreak of Omicron as an exclusive COVID-19 center. Oropharyngeal or nasopharyngeal swabs were collected during hospitalization for RT‐qPCR. According to the diagnosis and treatment protocol for COVID-19 (trial version 9) 7, all hospitalized patients were discharged after only two consecutive negative polymerase chain reaction (PCR) tests. Breakthrough infections were defined as laboratory-confirmed (PCR or rapid antigen test) SARS-CoV-2 infections that occurred at least 14 days after full vaccination. This study followed the principles of the Declaration of Helsinki and was approved by the Ethics Committee of the Southwest Hospital, Army Medical University (Third Military Medical University) (approval number: KY2022114).

### Data Sources

Demographic data (age and sex) and clinical parameters, including chronic comorbid conditions, clinical symptoms, nucleic acid Ct values, and time to viral clearance, were obtained from medical records and examined by two independent researchers. PCR expressed the viral load, and the test results and Ct values were recorded for each SARS-CoV-2 positive person. The Ct value reflects the number of cycles required to detect the viral genetic material during PCR amplification and is inversely proportional to the number of target nucleic acids in the test sample. The average values of the ORFLab gene and N gene-positive results of patients in Fangcang hospitals were used as the average viral load, and the lowest value was used as the highest viral load. The number of days from the first positive nucleic acid test to the first negative test (in two consecutive tests) was defined as the viral clearance time. The control variables used in this study included demographic data, vaccinations, and comorbidities. Symptoms in the early stages of infection were self-reported when mild and asymptomatic patients were admitted to Fangcang Hospital. The participants were stratified according to their symptoms, age, and sex. The following five age groups were identified: [0-18], [18-45], [45-60], [60-75], and > 75 years. All data are accessible through direct contacting with the corresponding author.

### Statistical Analysis

The social science statistical software package (SPSS, version 26.0; R language 4.2 were used for the statistical analysis. Statistical significance was set at p < 0.05. Normal continuous variables are summarized as mean ± standard deviation. Independent-sample t-tests were used to compare continuous data. Non-normally distributed continuous variables were expressed as medians and quartile ranges and compared using the Mann-Whitney U and Kruskal-Wallis H tests. Categorical variables were expressed as numbers and percentages, and comparisons were made using the χ^2^ test and Fisher's exact test. We used 1:1 PSM to match the baseline characteristics in the queue. The nearest-neighbor matching method was used for PSM matching to perform 1:1 matching. The standard caliper was set at 0.01. Propensity score matching was based on age, sex, vaccination status, and previous medical history (hypertension, diabetes, cardiovascular disease, cerebrovascular disease, respiratory disease, kidney disease, infectious disease, and tumor), with a standardized mean difference (SMD) of less than 0.1 indicates a good balance of PSM. Independent sample t-tests were used to compare the risk of different symptoms, nucleic acid CT values, and viral clearance times between the male and female vaccinated and unvaccinated groups and different age groups. All tests were two-tailed, and significance was set at a p-value lower than 0.05.

## Results

We enrolled 68191 Omicron patients with mild disease, including 26613 females and 41578 males. A total of 18023 (26.4%) patients had clinical symptoms upon admission, of which the most common were cough (12929, 18.9%) and expectoration (8351, 12.2%). Furthermore, 52429 (76.9%) patients were vaccinated, and 15762 (23.1%) were unvaccinated.

### Gender

**Figure 1:**
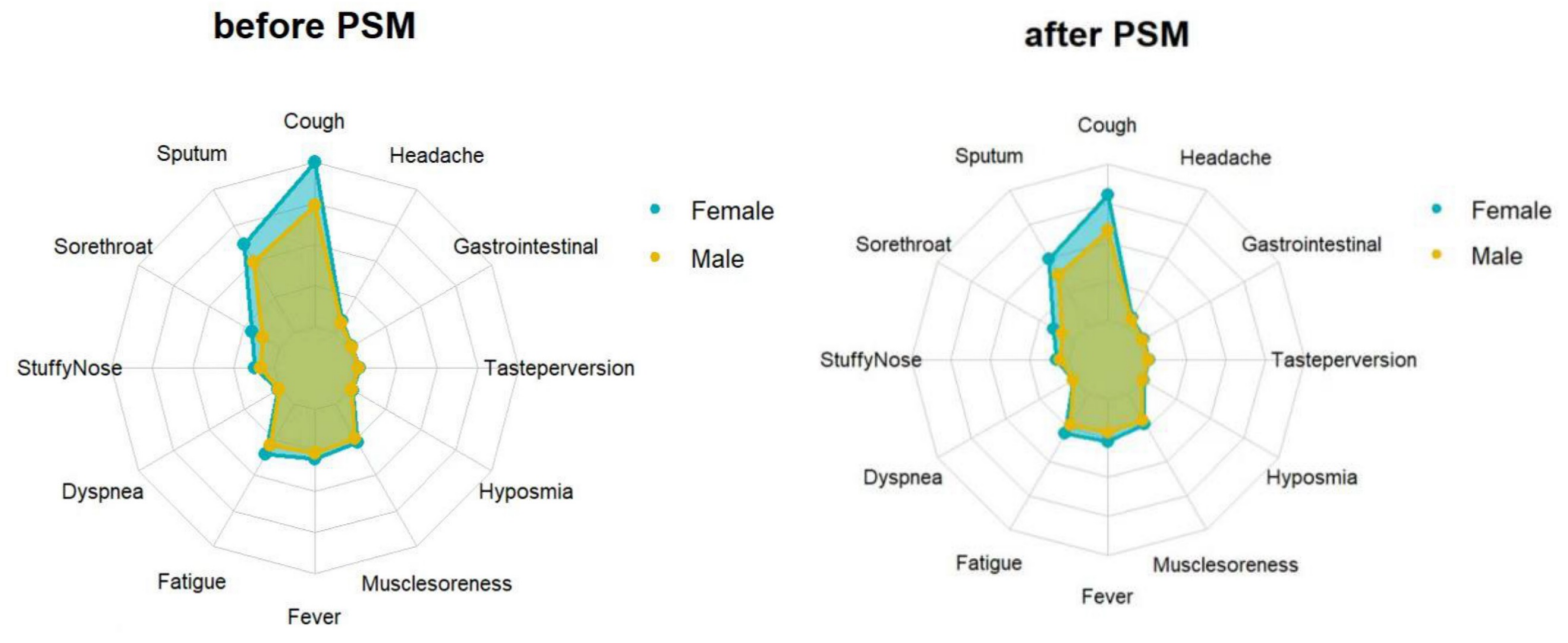
** Feature relevance by gender.** Symptoms are grouped according to gender: The yellow and blue line separately represents the symptom of males and females. Points further from the center correspond to a longer relevance.

The average viral clearance time in the male group was 5.41 days, which was longer than that in the female group (5.32 days) (*p* < 0.001). The mean and minimum values of the ORF1ab and N genes in the male group were longer than in the female group (32.71VS32.50, 30.71VS30.55, *p* < 0.001; 30.28VS30, 28.40VS28.16, *p* < 0.001) **(Table 1)**. The average and highest viral loads in the female group were significantly higher than those in the male group. There were differences in the clinical symptoms between men and women on admission. Specifically, 8169 (35.4%) of 26613 females and 9854 (23.7%) of 9854 males tested negative and reported having clinical symptoms. The most common symptoms were cough, expectoration, and fatigue, which were more common in women than males (all *p* < 0.001)** (Table 2) (Fig. 1).**

**Figure 2:**
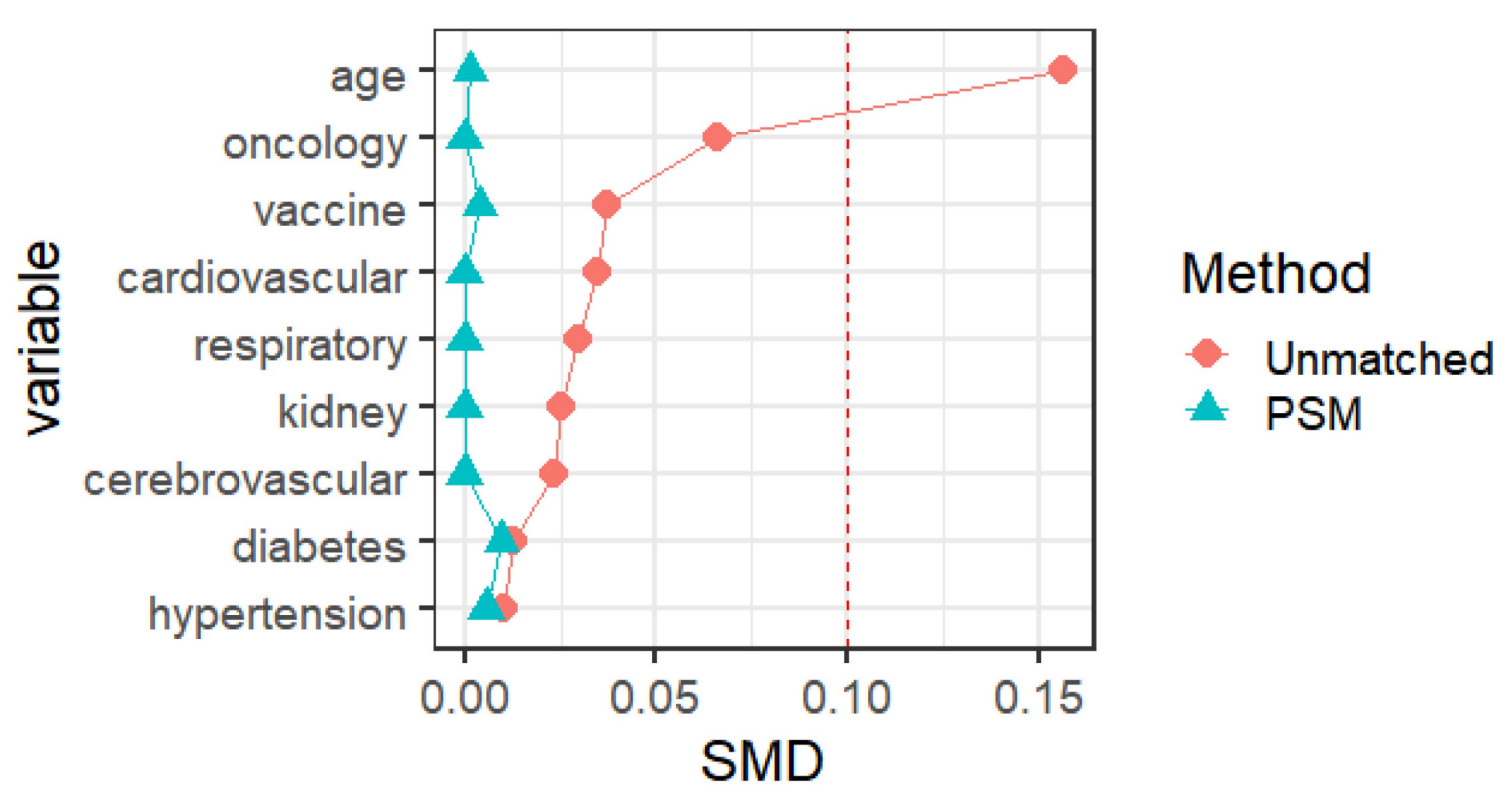
Baseline characteristics of COVID-19 patients male and female before and after PSM matching.

Propensity score matching identified 25827 pairs of patients (25827 females and 25827 males) according to age, comorbidities, and vaccination. Before PSM matching, the female and male groups differed significantly for all included variables, such as age, vaccination, and common fundamental diseases (*p* < 0.05). There were no significant differences in the baseline characteristics between the two groups, suggesting that each group was well-matched using a 1:1 matching algorithm (*p* < 0.05)** (Fig. 2)**. Moreover, we analyzed the differences between the female and male groups. At admission, there were no significant differences in viral clearance time between the two groups (*p* = 0.41). However, the median titers of the average viral load (ORF1ab and N gene) in the female group were 32.65 and 30.73, longer than those in the male group (p < 0.001), which were at 32.94 and 30.94, respectively. The highest viral loads (ORF1ab and N genes) were in the female group, with an average of 30.24 and 28.45, which were significantly higher than those in the male group (*p* < 0.001), at 30.65 and 28.84, respectively **(Table 1)**. In the early stages of infection, the proportion of cough, sputum production, fatigue, fever, muscle soreness, sore throat, nasal congestion, olfactory disorder, and taste disorder in the female group was significantly higher than that in the male group** (Table 2) (Fig. 1)** (*p* < 0.005).

### Age

**Figure 3:**
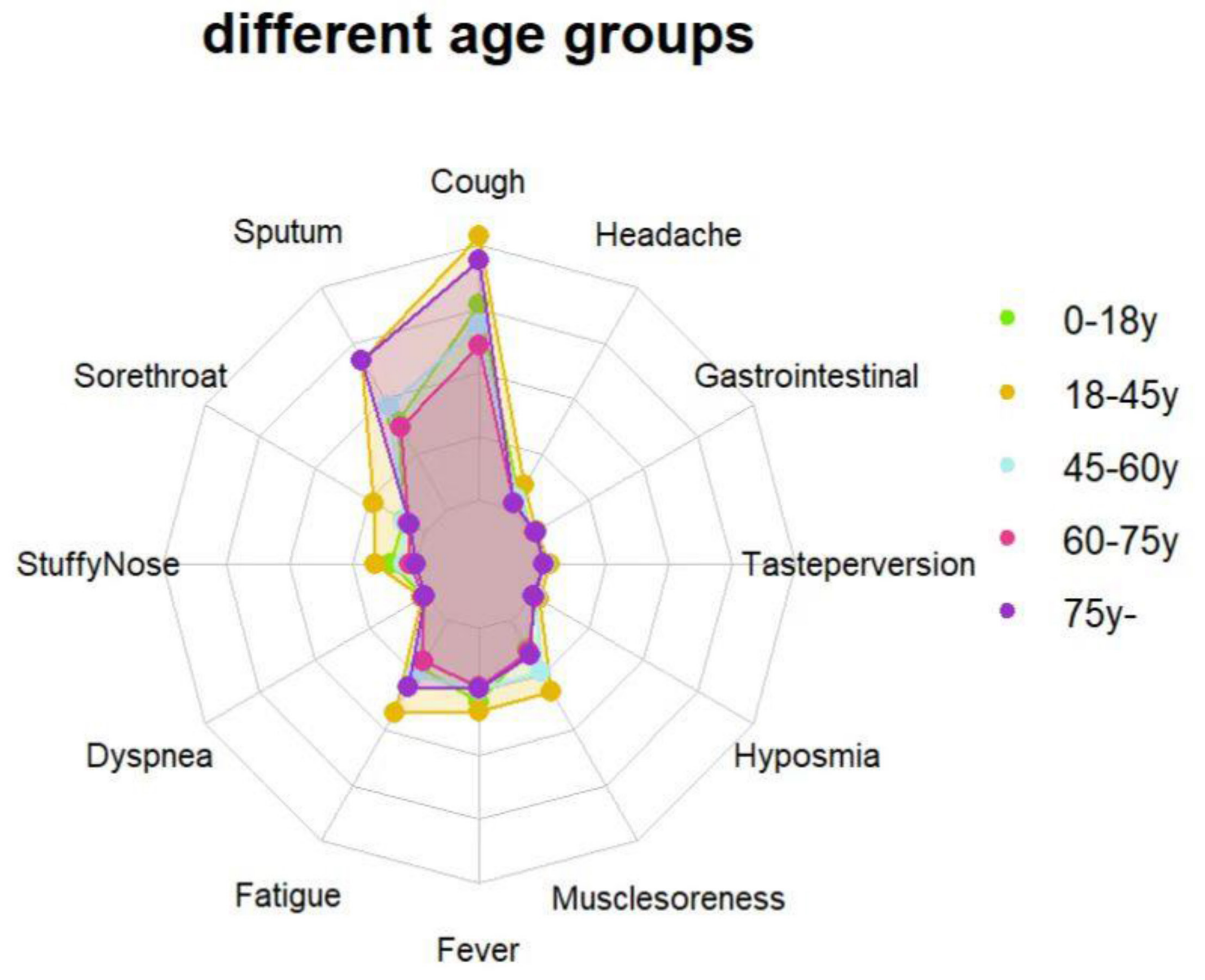
** Feature relevance by age groups.** Symptoms are grouped according to age: 0-18 years group (green line), 18-45 years group (yellow line), 45-60 years group (blue line), 60-75 years group (red line), over 75 years group (purple line). Points further from the center correspond to a longer relevance.

The average viral clearance time in the > 75 years group was the longest (6.64 days), which was significantly longer than that in the 18-45 years group (4.94 days) (*p* < 0.001). The average viral load and the highest viral load in the 60-75 years group were significantly higher than those in the other groups. The mean and minimum values of ORF1ab and N genes in the 60-75 years group were lower than those in the other age groups (29.13, 27.27, *p* < 0.001; 32.39, 30.26, *p* < 0.001)** (Table 3)**. There were differences in the clinical symptoms among the different age groups (*p* < 0.001). The 18-45 years old group had the most clinical symptoms on admission (45.4%), while those aged > 75 years had the least (0.7%)** (Table 4) (Fig. 3).**

### Chronic comorbid conditions

As **Supplementary Table 1**, the viral clearance time, average viral load, and highest viral load, in patients with comorbid chronic diseases were longer in those with hypertension, diabetes and cardiovascular diseases (all *p* < 0.05). but there was no statistically significant difference in cerebrovascular disease, respiratory diseases, kidney disease and tumors (all *p* > 0.05). In addition, there were significant differences in clinical symptoms between those with chronic diseases such as hypertension, diabetes, cardiovascular diseases, respiratory diseases and hypothyroidism (all *p* < 0.05), while there were no significant differences in tumor and cerebrovascular disease (all *p* > 0.05).

### Vaccine

**Figure 4:**
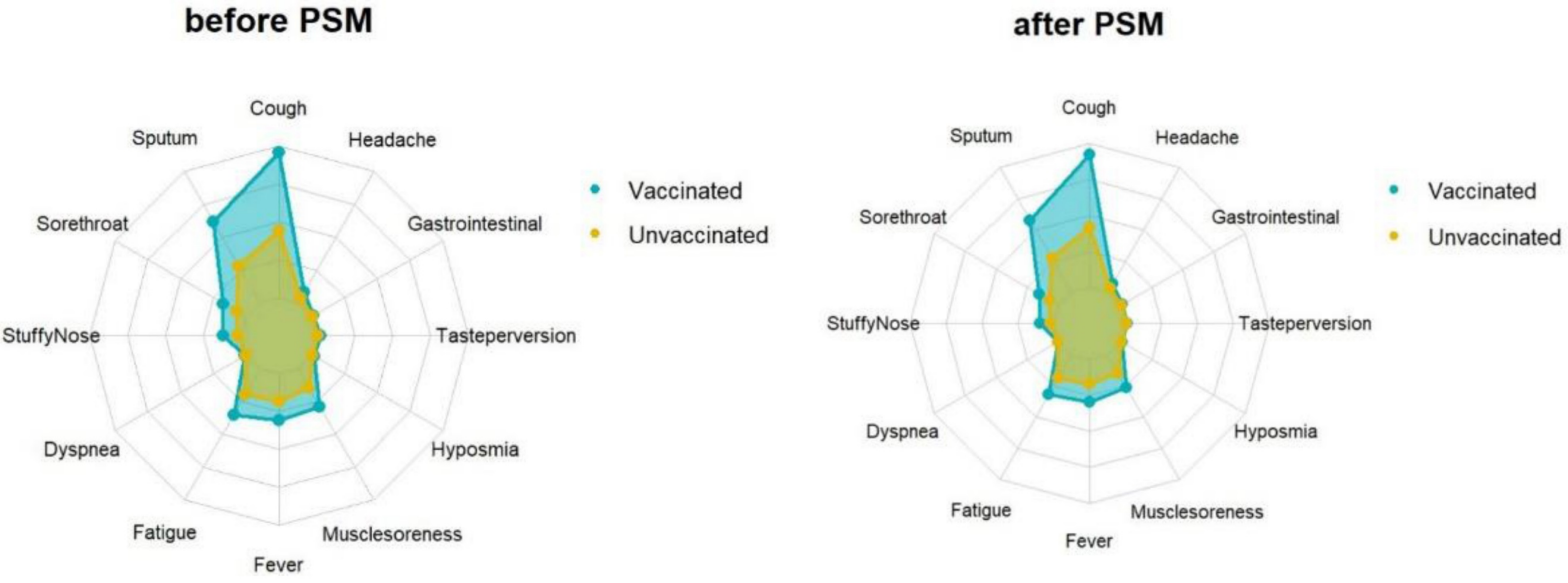
** Feature relevance by vaccine.** Symptoms are grouped according to vaccine: The yellow and blue line separately represents the symptom of vaccinated and unvaccinated. Points further from the center correspond to a longer relevance.

The average viral clearance time in the unvaccinated group was 5.82 days, significantly longer than in the vaccine breakthrough infection group, which was 5.24 days (*p* < 0.001). The mean values of the ORF1ab and N genes in the vaccine breakthrough infection group were longer than those in the unvaccinated groups (32.68 vs 32.44, 30.72 vs 30.45, *p* < 0.001). The minimum values of ORF1ab and N genes in the vaccine breakthrough infection groups were longer than those in the unvaccinated groups (30.32 vs 29.66, 28.46 vs 27.80, *p* < 0.001)** (Table 5)**. The average and highest viral loads in the unvaccinated group were longer than those in the vaccine breakthrough infection group. There were differences in the clinical symptoms between the vaccine breakthrough infections and the unvaccinated groups. The clinical symptoms in the vaccine breakthrough infection group were significantly more severe than those in the unvaccinated group (*p* < 0.001). Furthermore, cough sputum and fatigue were the most common symptoms in both **groups (Table 6) (Fig. 4)**.

**Figure 5:**
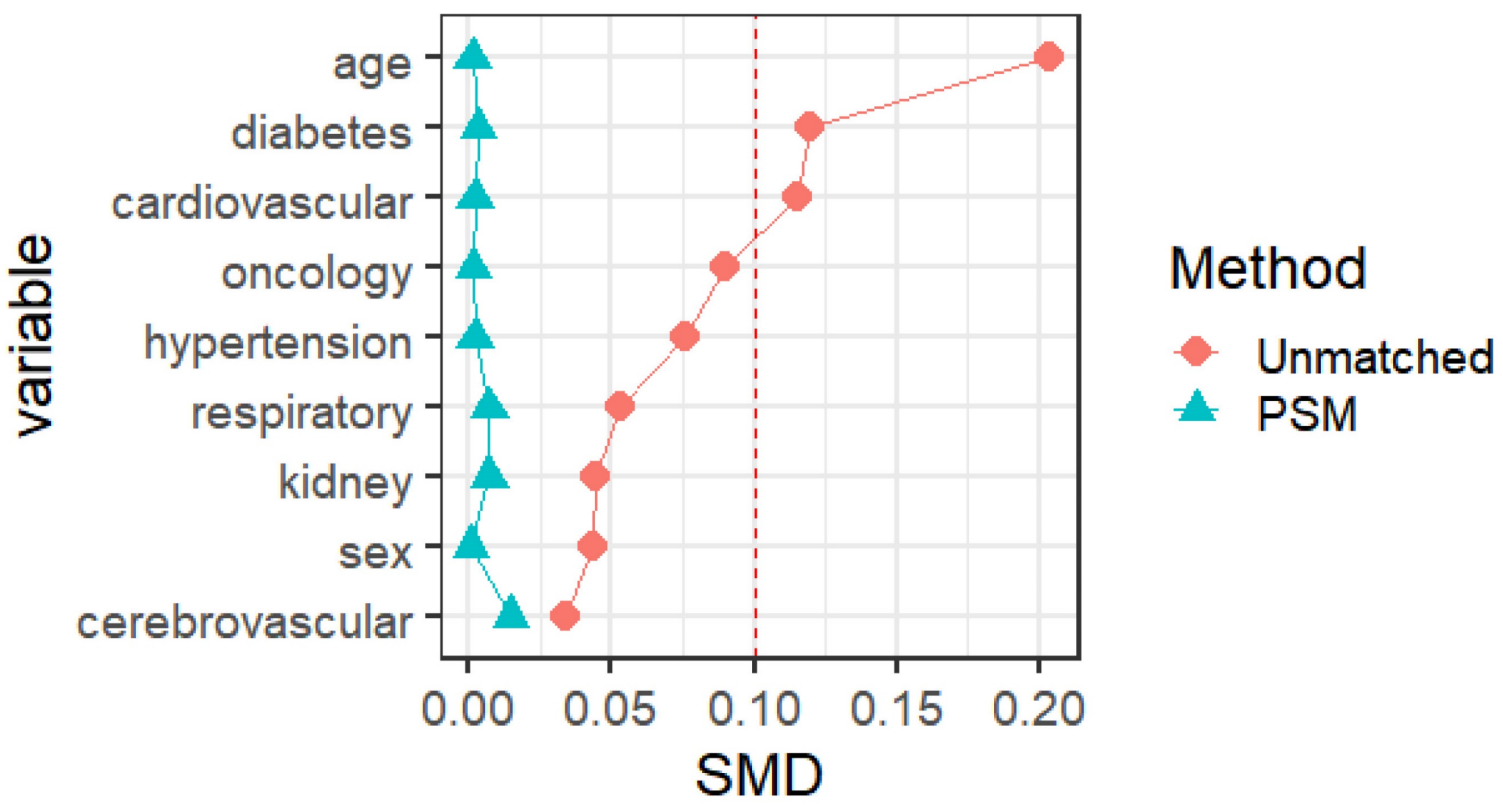
Baseline characteristics of COVID-19 patients vaccinated and unvaccinated before and after PSM matching.

Propensity score matching identified 14813 pairs of patients (14813 vaccinated and 14813 unvaccinated) according to age, sex, and comorbidities. Before PSM matching, the vaccinated and unvaccinated groups were significantly different for all included variables, such as age, sex, and common fundamental diseases (*p* < 0.05). There were no significant differences in the baseline characteristics between the two groups, suggesting that each group was well matched using a 1:1 matching algorithm (*p* < 0.05)** (Fig. 5)**. As shown in **Table 5**, the viral clearance time in the vaccine breakthrough infections group was significantly shorter (5.21VS5.78, p < 0.001), while the average viral load (ORF1ab gene: 32.80 vs 32.43. N gene: 30.78VS30.44. *p* < 0.01) and the highest viral load (ORF1ab gene: 30.55 vs 29.65, N gene: 28.64 vs 27.78, *p* < 0.01) were significantly lower than those in the unvaccinated group. In the early stages of infection, the proportion of cough, sputum production, fatigue, fever, muscle soreness, sore throat, nasal congestion, olfactory disorder, and taste disorder in the vaccine breakthrough infection group were significantly higher than those in the unvaccinated group (*p* < 0.005). Cough, phlegm, and fatigue were the most common symptoms **(Table 6) (Fig. 4)**.

## Discussion

Severe acute respiratory SARS-CoV-2 variant Omicron has caused panic reactions worldwide because of its high infectivity and vaccine immune escape mutations. It has been revealed that Omicron may be more than ten times more infectious than the original virus or about twice as contagious as the delta variant 3. Therefore, understanding the determinants of transmission, including human characteristics and vaccine effectiveness, is essential for developing preventive strategies and minimizing disease transmission, severity, and mortality. Based on the SARS-CoV-2 OmicronBA.2 in Shanghai in 2022, this study explored the relationship between vaccination, age, sex, clinical symptoms, days of nucleic acid-negative conversion, and viral load.

Age is an important risk factor for mortality of SARS-CoV-2. The most common clinical symptom in younger patients is a strong immune response, and most of them have been vaccinated, which enhances the immune response and quickly eliminates SARS-CoV-2; thus, the viral clearance time is the shortest. However, older individuals have a high viral load and long viral clearance time and are often complicated by hypertension, diabetes, and other diseases, resulting in delayed diagnosis and treatment and an aggravated prognosis of COVID-19^8^, ^9^. Hypertension, diabetes, cardiovascular disease, kidney disease, infectious diseases, tumors, and susceptibility conditions are associated with a longer risk of serious diseases or death. Due to the increased expression of angiotensin-converting enzyme 2 (ACE 2; receptors for coronavirus) in some concomitant diseases, such as hypertension and diabetes, SARS-CoV-2 enhances cellular attacks through ACE2 receptors 10.

Comorbidities increase the severity of COVID-19. In addition, there are fewer positive symptoms in the elderly, which is consistent with the results of previous studies 6, 11, 12. This may be attributed to older people having a lower immune response to vaccines, waning immunity, and other organ dysfunctions 13. Meanwhile, COVID-19 vaccines have a short duration of immune response and less efficacy in frail and older people, and many older people do not receive vaccinations 14. Therefore, while protecting the most vulnerable from infection, prevention, and control should focus on including the elderly or those with comorbidities and strengthening vaccination to reduce transmission and mortality.

We observed a significant difference between female and male COVID-19-positive individuals in the clinical symptoms and viral load. It is well known that males are generally more susceptible to infectious diseases than females. Epidemiological studies have shown that the morbidity and mortality rates in males are higher than in females. The risk of severe COVID-19 in male patients is 2.41 times longer than that in female patients 15. Clinically, there are differences in immune responses to SARS-CoV-2 between males and females due to various factors, including sex hormones and many immune genes, such as the longer expression of ACE 2 in males is longer than in females 16, 17. The viral load in males was longer than that in females due to the differential expression of ACE 2, the decrease in specific transcripts of B cells and NK cells, and the increase in androgens 15, 18, 19. In addition, the unhealthy lifestyle of men, including smoking and drinking, leads to high viral load and severity 20, 21. Furthermore, the irresponsible attitude among men for longer than females play an important role, such as frequent handwashing, wearing masks, and staying at home 20. However, this study showed that females have a longer SARS-CoV-2 viral load than men, but the viral clearance times in the two groups were similar. This may be because this study was based on mildly ill patients in a facing shelter hospital, whereas previous studies included all hospitalized patients with mild, severe, and critical illnesses 21. In addition, being sensitive to symptoms and better at expressing symptoms are reasons why women have more clinical symptoms. In sum, female COVID-19 patients in the study population had more symptoms than did their male counterparts.

Several studies have shown that SARS-CoV-2 mutants exhibit increased infectivity and virulence 2, 3. Additionally, some variants reduce the ability to neutralize the serum of vaccinated individuals 22. During the COVID-19 pandemic, the initial focus was on the elderly or those with preexisting health conditions, such as hypertension and diabetes, who were at a high risk of contracting COVID-19 and/or death 23. However, it is now clear that vaccination is also a contributing factor 24. Randomized clinical trials have demonstrated that vaccines against COVID-19 are highly efficacious (94%) 25. These vaccines effectively reduce the transmission and infectious complications of SARS-CoV-2 variants 26-28. It is reported that COVID‐19 vaccines still show an obvious advantage in reducing severe symptoms, hospitalization, and death. Moreover, vaccination accelerates viral clearance 2. However, no vaccine can entirely prevent the infection. Although FDA-authorized vaccines are highly effective, breakthroughs are still required 29. Due to weakened immunity and/or variant immune escape, the effectiveness of the COVID-19 vaccine in preventing COVID-19 has decreased, especially in the omicron-predominant period 4. The Omicron variant may be more susceptible to vaccine breakthrough infections than other variants 30. In addition, the vaccine's diminished efficacy and seasonal effects are likely to contribute to surges in post-vaccination infections. Therefore, with the continuous spread and variation in SARS-CoV-2, the emergence of various variants poses an increasing risk to global public health, especially breakthrough vaccine infections.

In China, breakthrough infections are occasionally detected despite strict prevention and high vaccination coverage rates. Our study summarized the clinical symptoms and features of COVID-19 vaccine breakthrough infections in Shanghai, China, from March 28, 2022, to June 28, 2022. The results showed that the symptoms of patients vaccinated with the COVID-19 vaccine increased significantly upon admission. Compared with patients without an inactivated vaccine, patients infected with SARS-CoV-2 after vaccination can rapidly produce antibodies in the early stages of the disease 17. The body has a longer immune response after exposure to the COVID-19 vaccine, and the symptoms are more severe when the virus invades again. However, the viral load and clearance time in patients with breakthrough infections were significantly lower than those in unvaccinated patients. These studies show that once breakthrough infections emerge, vaccination does not necessarily reduce the clinical symptoms, but it can shorten the viral load and the viral clearance time, which is also important for rehabilitating COVID‐19 and reducing transmission to others. In addition, booster vaccination is associated with a lower risk of breakthrough infections 27, 31. Overall, COVID-19 vaccines are effective in reducing disease severity 13. These findings demonstrated the feasibility and necessity of vaccination. However, a major limitation of actual COVID-19 vaccines is breakthrough infections, which should be enhanced for future improvements.

This study has several limitations. Firstly, this study was single-center, which limits the generalization of the study findings outside the regional interest, and we will conduct a multicenter study. In addition, most patients from the outbreaks described in this study had mild or no symptoms, but we did not consider patients with breakthrough infections and pneumonia, which might be a limitation of this study. What's more, we have not taken into account the impact of vaccine type, dosage, timing, and specific chronic diseases on COVID-19 in our analysis and it will be subject in subsequent research. Finally, we did not detect viral RNA in the urine or fecal specimens of the patients.

## Conclusion

In conclusion, our study summarized the clinical features of Omicron variant mild omicron variants and indicated that female patients, the elderly, and those with underlying comorbidities have longer clinically positive symptoms and viral loads. This will help patients make appropriate medical decisions based on age, sex, comorbidities, and clinical symptoms. Above all, although vaccination may not reduce clinical symptoms and block COVID-19 transmission, it can shorten the viral load and clearance time, which is also important for recovering from COVID‐19 and reducing transmission to others. In addition, booster vaccination should be considered in patients at major risk of complications.

## Supplementary Material

Supplementary table.

## Figures and Tables

**Table 1:** Associations of viral load and viral clearance time with the gender of COVID-19 patients.

Characters	Before PSM				After PSM			
	Female	Male	t	*p*	Female	Male	t	*p*
Average values of ORF1ab gene	32.5 (2.70)	32.71 (2.64)	-0.62	< 0.001	32.51 (2.70)	32.75 (2.66)	-10.39	< 0.001
Average values of N gene	30.55 (2.52)	30.71 (2.45)	-8.48	< 0.001	30.55 (2.52)	30.73 (2.46)	-8.19	< 0.001
Minimum values of ORF1ab gene	30.00 (4.30)	30.28 (4.20)	-8.47	< 0.001	30.01 (4.30)	30.39 (4.21)	-10.14	< 0.001
Minimum values of N gene	28.16 (4.11)	28.4 (4.01)	-7.48	< 0.001	28.18 (4.11)	28.48 (4.00)	-8.55	< 0.001
viral clearance time	5.32 (2.89)	5.41 (3.03)	-4.24	< 0.001	5.29 (2.87)	5.36 (3.00)	-2.94	0.003

Data are mean (SD).

**Table 2:** Differences in presenting clinical symptoms among COVID-19 patients by gender.

Characters	Before PSM				After PSM			
	Female/n (%)	Male/n (%)	t	*p*	Female	Male	t	*p*
N (%)	26613 (39.0)	41578 (61.0)			25827 (50.0)	25827(50.0)		
Positive symptoms	8169 (30.7)	9854 (23.7)	408.38	< 0.001	7867 (30.5)	5985 (23.2)	349.39	< 0.001
Cough	6007 (22.6)	6922 (16.7)	370.55	< 0.001	5778 (22.4)	4272 (16.5)	280.19	< 0.001
Sputum	3716 (13.9)	4633 (11.2)	120.12	< 0.001	3562 (13.8)	2818 (10.9)	98.99	< 0.001
Sore throat	1193 (4.5)	1129 (2.7)	154.10	< 0.001	1166 (4.5)	576 (2.2)	206.80	< 0.001
Stuffy nose	725 (2.7)	783 (1.9)	53.07	< 0.001	714 (2.8)	418 (1.6)	79.14	< 0.001
Dyspnea	45 (0.2)	57 (0.2)	1.11	0.29	42 (0.2)	29 (0.1)	2.39	0.123
Fatigue	2105 (7.9)	2736 (6.6)	43.47	< 0.001	2009 (7.8)	1608 (6.2)	47.81	< 0.001
Fever	1828 (6.9)	2474 (5.9)	23.16	< 0.001	1740 (6.7)	1478 (5.7)	22.75	< 0.001
Muscle soreness	1625 (6.1)	2301 (5.5)	9.78	< 0.001	1560 (6.0)	1408 (5.5)	8.26	0.004
Hyposmia	102 (0.4)	119 (0.3)	4.73	0.03	100 (0.4)	55 (0.2)	13.11	< 0.001
Taste perversion	131 (0.5)	150 (0.4)	6.84	0.01	128 (0.5)	74 (0.3)	14.49	< 0.001
Gatrointestinal	78 (0.3)	87 (0.2)	2.24	0.13	46 (0.2)	40 (0.2)	0.42	0.517
Headache	479 (1.8)	631 (1.5)	8.07	< 0.001	64 (0.2)	53 (0.2)	1.04	0.309

**Table 3:** Differences in viral load and viral clearance time by the age of COVID-19 patients.

Characters	< 18	18-45	45-60	60-75	≥ 75	F	*p*
Average values of ORF1ab gene	50 (1.42)	758 (2.45)	215 (1.04)	84 (0.67)	3 (0.61)	89.04	< 0.001
Average values of N gene	32.87 (2.65)	32.75 (2.71)	32.65 (2.61)	32.25 (2.60)	32.39 (2.59)	114.01	< 0.001
Minimum values of ORF1ab gene	30.96 (2.48)	30.77 (2.51)	30.65 (2.42)	30.26 (2.44)	30.43 (2.41)	283.27	< 0.001
Minimum values of N gene	30.84 (4.01)	30.55 (4.17)	30.13 (4.18)	29.13 (4.39)	29.34 (4.47)	313.43	< 0.001
Nucleic acid-negative days	29.03 (3.84)	28.69 (3.97)	28.25 (3.98)	27.27 (4.22)	27.55 (4.26)	568.02	< 0.001

**Table 4:** Differences in presenting clinical symptoms among COVID-19 patients by age.

Characters	< 18	18-45	45-60	60-75	≥ 75	F	*p*
	3531 (5.18)	30955 (45.39)	20696 (30.35)	12516 (18.35)	493 (0.72)		
Positive Symptoms	824 (23.34)	10042 (32.44)	4665 (22.54)	2360 (18.86)	132 (26.77)	1122.82	< 0.001
Cough	615 (17.42)	7242 (23.40)	3244 (15.67)	1723 (13.77)	105 (21.30)	768.64	< 0.001
Sputum	315 (8.92)	4725 (15.26)	2188 (10.57)	1046 (8.36)	75 (15.21)	532.92	< 0.001
Sore throat	75 (2.12)	1601 (5.17)	432 (2.09)	207 (1.65)	7 (1.42)	543.30	< 0.001
Stuffy nose	81 (2.29)	1134 (3.66)	221 (1.07)	72 (0.58)	0 (0.00)	593.13	< 0.001
Dyspnea	0 (0.00)	70 (0.23)	20 (0.10)	12 (0.10)	0 (0.00)	24.48	< 0.001
Fatigue	172 (4.87)	2934 (9.48)	1166 (5.63)	535 (4.27)	34 (6.90)	511.06	< 0.001
Fever	233 (6.60)	2275 (7.35)	1123 (5.43)	645 (5.15)	26 (5.27)	113.65	< 0.001
Muscle soreness	103 (2.92)	2279 (7.36)	1117 (5.40)	409 (3.27)	18 (3.65)	351.40	< 0.001
Hyposmia	7 (0.20)	162 (0.52)	40 (0.19)	12 (0.10)	0 (0.00)	72.52	< 0.001
Taste perversion	7 (0.20)	194 (0.63)	60 (0.29)	19 (0.15)	1 (0.20)	67.40	< 0.001
Gatrointestinal	4 (0.12)	105 (0.34)	42 (0.20)	13 (0.11)	1 (0.20)	14.87	< 0.001
Headache	50 (1.42)	758 (2.45)	215 (1.04)	84 (0.67)	3 (0.61)	250.83	< 0.001

**Table 5:** Differences in viral load and viral clearance time by the vaccinated status of COVID-19 patients.

Characters	Before PSM				After PSM			
	Vaccinated	Unvaccinated	t	*p*	Vaccinated	Unvaccinated	t	*p*
Average values of ORF1ab gene	32.68 (2.66)	32.44 (2.66)	-10.01	< 0.001	32.8 (2.63)	32.43 (2.66)	-11.91	< 0.001
Average values of N gene	30.71 (2.47)	30.45 (2.47)	-11.45	< 0.001	30.78 (2.43)	30.44 (2.47)	-11.94	< 0.001
Minimum values of ORF1ab gene	30.32 (4.20)	29.66 (4.35)	-16.83	< 0.001	30.55 (4.17)	29.65 (4.35)	-18.17	< 0.001
Minimum values of N gene	28.46 (4.01)	27.8 (4.16)	-17.70	< 0.001	28.64 (3.96)	27.78 (4.15)	-18.09	< 0.001
Nucleic acid-negative days	5.24 (2.87)	5.82 (3.26)	19.84	< 0.001	5.21 (2.85)	5.78 (3.21)	15.98	< 0.001

**Table 6:** Clinical presenting symptoms among vaccinated and unvaccinated COVID-19 patients.

Characters	Before PSM				After PSM			
	Vaccinated	Unvaccinated	t	*p*	Vaccinated	Unvaccinated	t	*p*
n (%)	52429 (76.9)	15762 (23.1)			14813 (50.0)	14813 (50.0)		
Positive Symptoms	15672 (29.9)	2351 (14.9)	1397.85	< 0.001	4267 (28.8)	2120 (14.3)	920.07	< 0.001
Cough	11359 (21.7)	1570 (10.0)	1080.56	< 0.001	3091 (20.9)	1403 (9.5)	747.41	< 0.001
Sputum	7340 (14.0)	1009 (6.4)	651.20	< 0.001	1935 (13.1)	915 (6.2)	403.91	< 0.001
Sore throat	2066 (4.0)	256 (1.6)	197.70	< 0.001	515 (3.5)	240 (1.6)	102.79	< 0.001
Stuffy Nose	1416 (2.7)	92 (0.6)	251.18	< 0.001	324 (2.2)	82 (0.6)	146.25	< 0.001
Dyspnea	89 (0.2)	13 (0.1)	6.18	0.010	22 (0.2)	12 (0.1)	2.95	0.09
Fatigue	4142 (7.9)	699 (4.4)	220.68	< 0.001	1070 (7.2)	632 (4.3)	119.59	< 0.001
Fever	3649 (7.0)	653 (4.2)	162.70	< 0.001	988 (6.7)	579 (3.9)	112.71	< 0.001
Muscle Soreness	3404 (6.5)	522 (3.3)	225.98	< 0.001	878 (5.9)	481 (3.3)	121.55	< 0.001
Hyposmia	197 (0.4)	24 (0.2)	18.74	< 0.001	34 (0.2)	24 (0.2)	1.73	0.19
Taste Perversion	254 (0.5)	27 (0.2)	28.96	< 0.001	49 (0.3)	26 (0.2)	7.07	0.01
Gatrointestinal	139 (0.3)	26 (0.2)	7.23	< 0.001	37 (0.3)	23 (0.2)	2.01	0.12
Headache	977 (1.9)	133 (0.9)	78.69	< 0.001	224 (1.5)	121 (0.8)	31.11	< 0.001

## Abbreviations

ACE 2: Angiotensin-converting enzyme 2
BA: Omicron BA.2 variant
COVID-19: Coronavirus disease 2019
Ct: Cycle threshold
FDA: Food and Drug Administration
NK: Natural killer
ORF: Open reading frame
PCR: Polymerase chain reaction
PSM: Propensity score matching
RT-qPCR: Reverse transcription quantitative polymerase chain reaction
SARS-CoV-2: severe acute respiratory syndrome coronavirus 2
SPSS: Social science statistical software
WHO: World Health Organization
